# Band gap engineering of In(Ga)N/GaN short period superlattices

**DOI:** 10.1038/s41598-017-16022-z

**Published:** 2017-11-22

**Authors:** I. Gorczyca, T. Suski, P. Strak, G. Staszczak, N. E. Christensen

**Affiliations:** 1 Institute of High Pressures Physics, UNIPRESS, 01-142 Warsaw, Poland; 2 Department of Physics and Astronomy, Aarhus University, DK-8000 Aarhus C, Denmark

## Abstract

Discussion of band gap behavior based on first principles calculations of the electronic band structures for several InN/GaN superlattices (SLs) (free-standing and pseudomorphic) grown along different directions (polar and nonpolar) is presented. Taking into account the dependence on internal strain and lattice geometry mainly two factors influence the dependence of the band gap, *E*_*g*_ on the layer thickness: the internal electric field and the hyb wells) is more important. We also consider mIn ridization of well and barrier wave functions. We illustrate their influence on the band gap engineering by calculating the strength of built-in electric field and the oscillator strength. It appears that there are two interesting ranges of layer thicknesses. In one the influence of the electric field on the gaps is dominant (wider wells), whereas in the other the wave function hybridization (narrow wells) is more important. We also consider *m*In_*0*.*33*_Ga_*0.67*_N/*n*GaN SLs, which seem to be easier to fabricate than high In content quantum wells. The calculated band gaps are compared with recent experimental data. It is shown that for In(Ga)N/GaN superlattices it is possible to exceed by far the range of band gap values, which can be realized in ternary InGaN alloys.

## Introduction

During several years the properties of InGaN/GaN and other nitride short period SLs have been intensively studied. Many publications describe various aspects of In(Ga)N/GaN SLs, from epitaxial growth details, to band gap values in optoelectronic devices with In(Ga)N/GaN as the active region^1–5^. In particular, several of these works were devoted to the study of the mechanisms of radiative recombination in the SLs. According to theory, the short period (few atomic layers) *m*In(Ga)N/*n*GaN SLs, where *m* and *n* represent the numbers of atomic monolayers (MLs) make it possible to tune the band gap over a large range in the visible and UV spectrum. This is realized by varying the quantum well (QW) and quantum barrier (QB) layer thicknesses.

The pioneering work by Yoshikawa *et al*.^1^ initiated the interest in binary InN/GaN. It appeared as an idea to solve the difficulties in preparation of uniform In_*x*_Ga_*1−x*_N alloys with tunable chemical composition and thus band gap values *E*_g_. It is well known that In_*x*_Ga_*1−x*_N alloys with high *x* exhibit a phase separation introducing macroscopic non-uniformities in In_*x*_Ga_*1−x*_N for *x* > 0.25. The *m*InN/*n*GaN SLs seemed to be very attractive to replace In_*x*_Ga_*1−x*_N alloys with high *x*. Due to the limited amount of experimental data on InN/GaN SLs due to difficulties in the epitaxial growth of these SLs many papers concentrate on theoretical considerations of their electronic band structure and structural properties. Band gap engineering, i.e. “tayloring” of the SL band gaps by varying the layer thicknesses (*m* and *n*), is crucial for the design of optoelectronic devices.

To realize band gap engineering in the polar InGaN system (i.e., grown along the wurtzite c-axis) it is important to analyse and understand all the factors influencing the band gap behaviour. In particular, the built-in electric field originating from the macroscopic polarization, the wave function hybridization, the internal strain caused by lattice mismatch between well and barrier layers, and the effect of the lattice geometry, i.e., the arrangements of In and Ga cations. In this work, based on first principles calculations, we consider the contributions of all these factors to the band gap engineering. To answer the question how the internal strain influences the E_g_ values, two cases of growth conditions are compared: the pseudomorphic (a-lattice constant of In(Ga)N matches to GaN) and free-standing (a-lattice constant of the SL is an average of In(Ga)N and GaN). By comparing band gaps in SLs grown along different directions of the wurtzite structure we demonstrate the effects of lattice geometry. Further, it will be demonstrated that apart from the factors mentioned above, the band gaps are influenced mainly by: the built-in electric field, E_el_ (in polar structures) and the wave function hybridization. The picture presented is somewhat simplified, but we believe, that it can describe rather well the main trends in the SL band gap behavior, qualitatively and quantitatively. Firstly, we discuss the E_g_ evolution in polar SLs, free-standing and pseudomorphically grown, and we compare it with the gap dependence on layer thickness in nonpolar SL structures without E_el_. Then, the contributions from the internal electric field and wave function hybridization are evaluated. It is demonstrated that the effect of wave function hybridization is dominant for narrow wells and barriers, whereas for wider layers in polar SLs influence of the internal electric field is more important.

It is shown that the creation of In(Ga)N/GaN superlattices makes it possible to go far beyond the range of *E*_*g*_ evalues realized in ternary InGaN alloys. In particular, for a given equivalent In-content a wide range of *E*_*g*_ tunability can be achieved including even closure of the band gap. In order to apply our analysis to the experimental situation related to difficulties in growing binary InN/GaN SLs we consider also In_*x*_Ga_*1−x*_N/GaN SLs with In content, *x* = 0.33, which seems to be currently the upper limit of In content in QWs of these SLs^6,7^. We compare results of band gap calculations for In_*0.33*_Ga_*0.67*_N/GaN SLs with the recent experimental photoluminescence (PL) data.

The present work is somewhat related to earlier research^6,8–11^, but to get a complete picture of the band gap engineering in In(Ga)N/GaN SLs new way of results presentation is applied and new sets of calculations have been performed. Evolution of the band gaps and electric fields in our previous works^6,8–11^ was illustrated and discussed in the context of effective cation concentration. Such a choice was motivated by the concept of comparison with corresponding alloys. In the present work we decided to perform such discussion in the context of number of barrier and well MLs. Such approach is based on intuitive understanding and enables to expose main microscopic mechanisms leading to SL formation. The new way of presentation of the results makes easier to analyse in detail all the factors influencing the band gap behavior. Moreover, we include now a study of the role of wave function overlap. It takes into account both electron-hole and well-barrier contributions. The oscillator strength is calculated and discussed. In the calculations of the band gaps and electric field magnitude the range of the structures (number of well and barrier MLs) is significantly increased. New conclusions are drawn pointing out on the dominant role of internal electric field and wave functions hybridization. Influence of the above mechanisms on the band gap behavior is illustrated quantitatively. Comparison between ‘binary’ InN/GaN and ‘ternary’ InGaN/GaN SLs shows much weaker effects of electric field contributions to the band gap reduction in the latter case.

## Results and Discussion

### InN/GaN SLs

#### Band gaps

**Figure 1:**
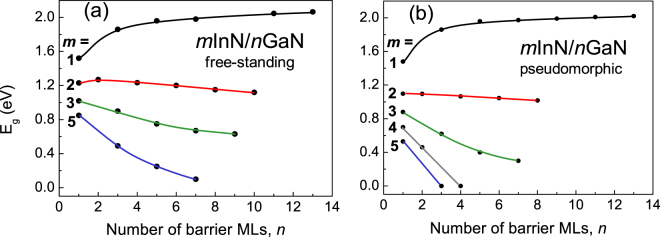
Calculated band gaps, *E*_*g*_, for *m*InN/*n*GaN SLs vs. number of barrier MLs for the free-standing (**a**) and pseudomorphic growth mode (**b**).

The band gap engineering, i.e. dependence of the band gaps on the thickness of the layers will be discussed first on the example of polar (grown along c –axis of the wurtzite structure) *m*InN/*n*GaN short period SLs. The simpler notation: *m/n* will often be used. Most of the calculations is performed for the case in which the SL is grown pseudomorphically on GaN substrate, thus having fixed in-plane lattice constants equal to the lattice constants of the unstrained GaN and the relaxation of the SL geometry is performed along the growth direction. To obtain high quality material the In(Ga)N/GaN structures are often grown on bulk GaN substrate. However, to illustrate the effect of strain we also performed band structure calculations for the free-standing structure, which involves a full relaxation of the lattice constants and internal parameters. In Fig. 1 the calculated band gaps versus layer thicknesses for sets of *m*InN/*n*GaN SLs are presented in a free-standing (Fig. 1a) and pseudomorphic (Fig. 1b) strain mode. Comparing both cases it is seen that all the trends in gap behavior are the same, but the *E*_*g*_ values are generally smaller in the pseudomorphic mode. In the case of 1/*n* SLs the difference is very small, but becoming larger for thicker wells, and it is quite pronounced for 5/*n* SLs. It reflects the influence of strain coming from the InN-GaN lattice mismatch on the InN layers, which causes the increasing degree of atomic relaxation along the growth direction.

Analysing the SL band gap dependence on the well and the barrier thickness we observe, that the band gaps are more sensitive to the well thickness than to the barrier width. *E*_*g*_ decreases rapidly with increasing well thickness, and this dependence is stronger for larger *n* values. Regarding the dependence on barrier thickness *E*_*g*_ in SLs with the 1 ML of InN well (*m* = 1) increases at first rapidly (up to *n* = 5), then slowly with increasing barrier thickness. In contrast, for SLs with more than one InN ML the band gap decreases with increasing well and barrier thickness. Band gaps smaller than *E*_*g*_ of pure InN (0.65 eV) occur in several cases from *m* > 3 (free-standing) and from *m* ≥ 3 (pseudomorphic). The metallization (closing of the effective band gap) occurs for *m* = *n* > 5 in the free standing mode (Fig. 1a) and already for *m* = *n* ≥ 4 in the pseudomorphic case (Fig. 1b). The latter effect was also demonstrated by Miao *et al*.^12^ and topological-insulator related aspects were pointed out.

The fact, that all the trends in gaps behavior are the same independently on the built-in strain (compare Fig. 1a,b) enable us to separate the strain effect from further discussion. Now, for given strain mode (usually pseudomorphic) the band gap evolution may be understood in terms of two counteracting effects: i) the hybridization of well and barrier wave functions and ii) the internal electric fields. The overall SL band gap corresponds to the local *E*_*g*_ of the InN ML. It emerges that the gaps in thin well SLs are dominated by the hybridization effect, which leads to a larger gap, due to the influence of the GaN-like wave functions on the states in the InN well. In 1/*n* SLs contributions to the InN well wave functions coming from neighbouring GaN layers cause a significant increase of the local gap from the value 0.65 eV (pertaining to bulk InN) to about 2.1 eV in the InN layer in the SL. Strong influence of the GaN-like wave functions on the states related to the InN well can be seen for up to *n* = 5, then *E*_*g*_ is almost constant, increasing very slowly. On the other hand, for wider wells the effect of the internal field dominates leading to the reduction of the *E*_*g*_ values. The internal electric fields lead to the spatial separation of electrons and holes, influencing strongly the band profiles along the growth direction and cause the band gaps to be “indirect in real space” and reduced in size, and eventually closing the gap. Reduced overlap of the electron-hole wave functions lower their radiative recombination rates and, accordingly, the efficiency of optoelectronic devices, both laser diodes LDs and light emiting diodes LEDs. A red-shift of the emitted light, i.e., the Quantum Confined Stark Effect, is observed.

**Figure 2:**
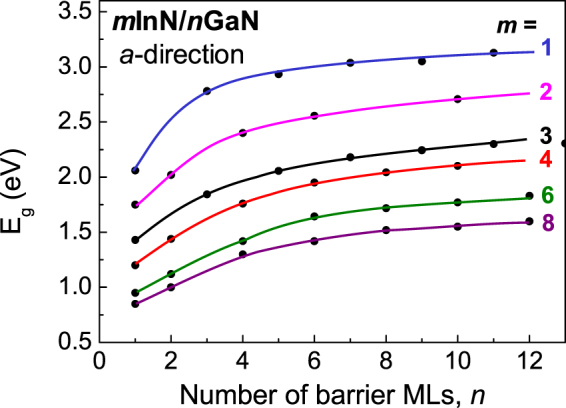
Calculated band gaps, *E*_*g*_, for *m*InN/*n*GaN SLs grown along the *a* < 11–20 > direction vs. number of barrier MLs.

One way to eliminate the built-in electric field is to grow the quantum-well structures and related emitters along the nonpolar *m* or *a* directions. Growing interest within the nitride community in the properties of these structures is observed. The calculated band gaps versus layer thicknesses for sets of nonpolar *m*InN/*n*GaN SLs grown along the *a* < 11–20 > direction are presented in Fig. 2. Figure 2 shows that in the absence of the electric field character of the *E*_*g*_ dependence on barrier thickness, *n*, is the same for all the *m* values – *E*_*g*_ increases with *n* rapidly up to *n* = 5, then more slowly reflecting decreasing influence of the wave functions hybridization. We observe also that *E*_*g*_ decreases with increasing well thickness, *m*, for all widths of barrier, *n*, but more slowly for higher *m* values reaching for 8/1 SL a slightly lower band gap (~0.5 eV) than that of InN (0.65 eV), which may reflect specific feature of SLs: the InN layer is strained to match the lattice constant of the GaN substrate.

#### Internal electric fields

To evaluate the influence of the electric field on the band gap behaviour we have to calculate its strength as a function of layer thickness. To do this we use the model described in the Section Methods. The advantage of the method described to estimate the electric field is, that it readily provides the electric field strengths for any values of the well and barrier thicknesses, whereas by ab-initio calculations we cannot obtain the internal electric field values for very thin and for very thick layers, due to computational restrictions. On the other hand, the model based on the parameters of the bulk materials constituting the SL neglects specific features of the SL and electric fields depend only on the effective chemical composition (*m/n* ratio), as follows from Eqs (5) and (6)), but not on the separate values of *m* and *n*. However, we can see from the comparison presented in ref.^13^ that the agreement between estimated and ab-initio calculated values of *E*_*w*_ and *E*_*b*_ is quite satisfactory.

**Figure 3:**
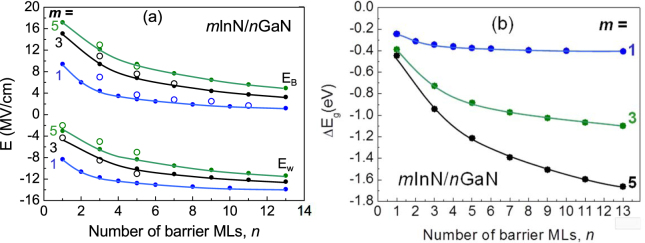
(**a**) Internal electric fields in wells and barrier of InN/GaN SL as functions of number of barrier MLs. Results of the model calculations (dots with lines) are compared for some cases with the ab-initio calculated fields (open circles). (**b**) Energy gap shift, *ΔE*_*g1*_, due to the internal electric field.

The electric fields obtained for polar *m*InN/*n*GaN SLs are illustrated on Fig. 3a. Series of constant *m* are traced with connecting lines for the well and the barrier. Results of the model calculations are compared with the ab-initio calculation for some structures. We can see that the agreement is quite good.

It is revealed that the absolute values of internal electric fields on the well side are increasing as function of barrier thickness at first rapidly, then starting from *n* = 5 more slowly, being almost constant for larger *n*, especially in the case of *m* = 1. In contrast, on the barrier side the internal electric field is decreasing with *n*, with the same character of this dependence as in a well. The variation between the constant-*m* series is relatively small on the well side and larger on the barrier side.

Generally, the SL band gap may be decomposed as:

1 $${E}_{g}(SL)={E}_{g}(well)+\Delta {E}_{g1}+\Delta {E}_{g2},$$

where *E*_*g*_ *(well)* denotes the band gap of the bulk well material. The influence of the electric field on the band gap depends on the strength of the electric field and on the well width being greater for wider wells. *ΔE*_*g1*_ is the total shift of the band edges across the well due to the internal electric field:

2 $$\Delta {E}_{g1}=eE{d}_{w}$$

*ΔE*_*g2*_ is the rest, i.e. including the effects of hybridization of well and barrier wave functions, and also local atomic relaxations and strains from the substrate matching. *ΔE*_g1_ as a function of barrier thickness is illustrated for different SLs on Fig. 3b. We observe that the influence of the electric fields can explain the lowering of the SL band gaps for wider wells.

**Figure 4:**
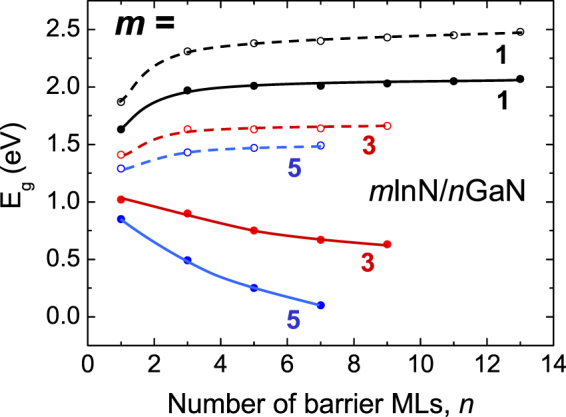
Calculated band gaps, *E*_*g*_ *(SL)*, for three sets*: 1*/n, 3/*n* and 5/*n* of *m*InN/*n*GaN SLs (solid lines) in comparison with estimated gaps *E*_*g*_ *(hyp)* (dashed lines) with the eliminated effect of the internal electric field (see text for discussion).

Having determined *ΔE*_*g1*_ allows us to compare the calculated band gaps (Fig. 1) of InN/GaN SL with the gaps for the hypothetical case that the internal electric field is “switched off”, i.e. *E*_*g*_ *(hyp)* = *E*_*g*_ *(SL)* − *ΔE*_g1_. Figure 4 illustrates the band gaps *E*_*g*_ *(hyp)* for the three sets of 1/*n*, 3/*n* and 5/*n* SLs in InN/GaN. Comparing with *E*_*g*_ *(SL)* the *E*_*g*_ *(hyp)* gaps are larger and show an increasing trend as function of the number *n* of barrier layers. It is shown that the band gaps of polar InN/GaN SLs when eliminated for the internal field effect would lie closer to the band gaps of nonpolar InN/GaN SLs. However, although the trends are very similar, the gap values are still different (larger in case of nonpolar SLs). Hence we may conclude that the difference between the gap trends in polar and nonpolar InN/GaN SLs are mainly due to the internal fields in the InN wells of SLs, but the effect of different lattice geometry should be also taken into account. The dependence on lattice geometry was discussed in ref.^10^ by comparing nonpolar SLs grown along different directions of the wurtzite structure (*a* and *m*).

As a numerical example, let us consider first the *1/1* SL where we have *E*_*g*_ *(SL)* = *1*.*63* eV, *E*_*g*_ *(InN)* = *0*.*65* eV, *ΔE*_*g1*_ = *−0*.*24* eV, and hence *ΔE*_*g2*_ = +*1*.*22* eV, what means that strain and hybridization effect is dominant in this case. Analogically, for *1*/*13* SL: *E*_*g*_ *(SL)* = *2*.*07 *eV, *ΔE*_*g1*_ = *−0*.*41* eV, *ΔE*_*g2*_ = +*0*.*83* eV, and still effect of electric field is weak. But, for *5/1* InN/GaN SL, *E*_*g*_ *(SL)* = *0*.*85 *eV, *ΔE*_*g1*_ = −*0*.*44* eV, and hence *ΔE*_*g2*_ = +*0*.*64* eV the difference between this two effects is smaller. Finally, for *5/7* InN/GaN SL, *E*_*g*_ *(SL)* = *0*.*1 *eV, *ΔE*_*g1*_ = −*1*.*39* eV, and *ΔE*_*g2*_ = +*0*.*84* eV. In this latter case the contribution from the electric field dominates over that from the strain and hybridization effect (strain in this case seems to be more important than hybridization). Concluding, for narrow wells the effect of strain and hybridization is dominant (effect of strain being weak for very narrow wells), whereas electric field effect is dominant for wide wells and barriers, e.g. for example for 5/7 SL it is almost 1.5 eV.

#### Oscillator strength

Both effects, overlap reduction between hole and electron states caused by the presence of electric field and weakening of the wave functions hybridization with increasing barrier thickness decrease not only the *E*_*g*_ values, i.e., PL energy emission, but also the PL intensity. The latter can be expressed by the overlap integral between the electron and hole wave functions, square of which reflects the oscillator strength (OS) of a band-to-band transition. Experimentally, it is related to the intensity of absorption and PL.

**Figure 5:**
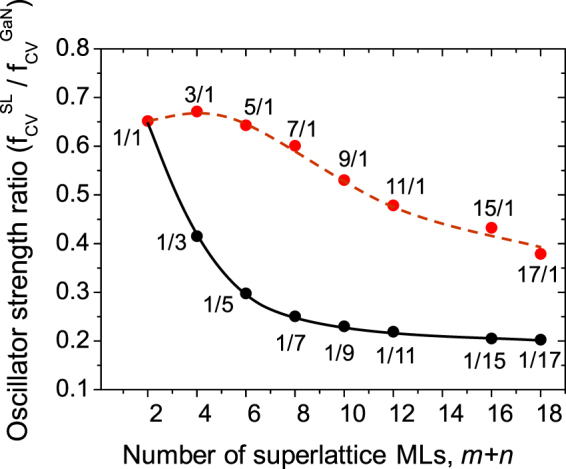
Oscillator strength (OS) ratio of SL and bulk GaN as a function of SL period thickness.

Figure 5 shows the ratio of transition matrix elements of edge transitions for SL and bulk GaN. The oscillator strength values for different structures were obtained from an implementation of the Projector Augmented Wave (PAW) method^14^ in an existing plane-wave code supporting non norm-conserving Vanderbilt-type ultra-soft pseudopotentials^15^, the Vienna ab initio simulation package VASP^16^. Based on the corresponding PAW-derived all electron wave functions, an implementation of the optical matrix elements in the VASP package is developed. The optical transition matrix elements are given by:

3 $${f}_{ij}=\frac{2{m}_{e}}{3\hslash }({ {\mathcal E} }_{j}-{ {\mathcal E} }_{i})\sum _{\alpha =x,y,z}{|\langle {\tilde{\psi }}_{i}|{\hat{R}}_{\alpha }|{\tilde{\psi }}_{j}\rangle |}^{2}$$

where ${ {\mathcal E} }_{i},{ {\mathcal E} }_{j}$ are the single-particle energies, *m*_*e*_ is the mass of an electron, ℏ is the reduced Planck constant, ${\tilde{\psi }}_{j}$, ${\tilde{\psi }}_{i}$ are the conduction and the valence wavefunctions, respectively, and ${\hat{R}}_{\alpha }$ is the position operator. In this formulation excitonic effects are neglected. The details of this model can be found in^17^.

As one can see on Fig. 5 the highest electron-hole transition probability, i.e., OS, is found for the set SLs with the shortest barrier, *n* = 1. OS is almost the same for very thin wells, but from *m* = 5 starts to decrease. For thin wells it can be explained by a weak effect of the electric field and strong well-barrier wave functions hybridization, whereas for thicker wells influence of the electric field is dominant reducing the OS. On the other hand, considering SLs with the single ML in the well (*m* = 1), with increasing barrier thickness a strong reduction of the wave functions hybridization occurs. It causes rapid reduction of the OS at the beginning, and then for thicker barriers, starting from around *n* = 5, the transition rates show tendency to saturate (around *n* = 15 the oscillator strength is around 20% of the bulk GaN value). This saturation results from the finite penetration lengths of electron and hole states into the SL barrier (an area in the GaN barrier begins to emerge wherein overlap between hole and electron states is approximately zero).

### SLs containing InGaN alloys

In the following we discuss the band gaps of short period *m*In_*x*_Ga_*1−x*_N/*n*GaN SLs grown along the wurtzite *c* axis. We choose In content, *x* = 0.33 in order to compare our calculated band gaps with the recent experimental PL data^18^. This composition seems to be currently the upper limit of experimentally achievable In content in QWs of *m*In_*x*_Ga_*1−x*_N/*n*GaN SLs^7^.

**Figure 6:**
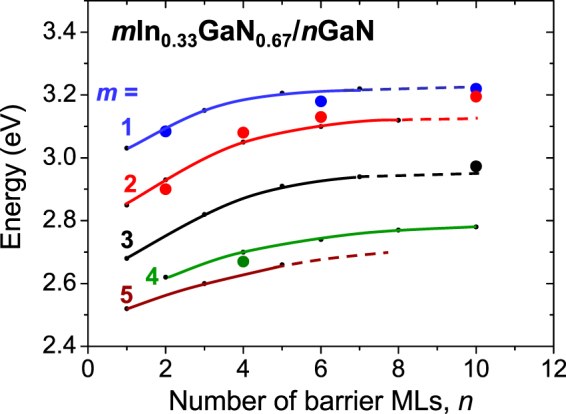
Calculated band gaps, *E*_*g*_, for *m*In_033_Ga_*0*.*67*_N/*n*GaN SLs vs. number of barrier MLs, *n*, in comparison with experimental PL data obtained for *m*In_*x*_Ga_*1−x*_N/*n*GaN samples with estimated *x* in the range: 0.30–0.33, blue dots correspond to *m* = 1, red dots are for *m* = 2, and green dot for *m* = 4.

Figure 6 shows the calculated band gaps of *m*In_033_Ga_*0*.*67*_N/*n*GaN, *E*_*g*_, vs. barrier thickness for different values of *m*. Experimental PL emission energies recently obtained on samples with different layer thicknesses are indicated by dots and we observe quite good agreement with the calculated gaps. Analysing Fig. 6 one can observe that the band gaps increase with increasing barrier thickness for all the considered well widths (up to 5). Contrary to the case of binary *m*InN/*n*GaN SLs, we do not observe the change of the increasing trend to decreasing one, however *E*_*g*_ increases more slowly for higher *m* values. Somehow the band gap behavior is intermediate between polar and nonpolar InN/GaN case and we can interpret it in terms of much weaker built electric field, which is sensitive to indium content in QW. Higher In content leads to higher lattice mismatch between InGaN and GaN layers what increases the piezoelectric polarization and consequently the electric field values.

**Figure 7:**
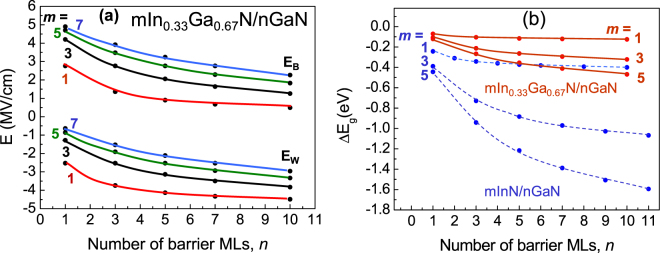
(**a**) Electric fields in wells (*E*_*w*_) and barriers (*E*_*B*_) of *m*In_*0*.*33*_Ga_0.67_N/*n*GaN SLs as functions of QB thickness. (**b**) Energy gap shift, *ΔE*_*g*1_, due to the internal electric field in *m*In_*0*.*33*_Ga_*0.67*_N/*n*GaN SLs (red solid lines) in comparison with *m*InN/*n*GaN SLs (blue dashed lines).

Internal electric fields in 1In_*0*.*33*_Ga_*0.67*_N/*n*GaN SLs, as obtained from the model are illustrated in Fig. 7a. Values of electric fields, *E*_*w*_ in wells, and *E*_*B*_ in barriers are given as functions of the QB thickness, *n*. Considering *E*_*w*_ and *E*_*B*_ dependence on *n*, one can distinguish two regions: thin QBs (*n* up to ~4 MLs) and thicker QBs (*n* > 5 MLs). In the 1^st^ region absolute values of electric field strongly increase with *n* in QW and strongly decrease in QB illustrating decreasing degree of QW wave functions penetration into the barriers. Then, in the 2^nd^ region the dependence of electric field on *n* becomes quite weak, as the coupling of wave functions between QWs is strongly reduced, and for thick enough barriers QWs can be treated as independent ones.

The influence of the electric fields can explain the lowering of the SL band gaps for wider wells, as illustrated on Fig. 7b. Comparing InN/GaN SLs with *m*In_*0*.*33*_Ga_*0.67*_N/*n*GaN SLs we observe on Fig. 7b, that the influence of the electric field on the band gap values is significantly weaker in the latter case. The primary cause for this effect is considerably smaller lattice mismatch in *m*In_*0*.*33*_Ga_*0.67*_N/*n*GaN SLs.

**Figure 8:**
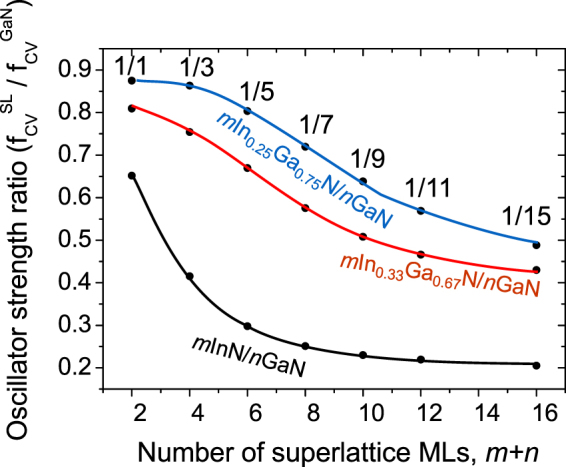
Oscillator strength ratio of SL and bulk GaN as a function of SL period thickness for different structures of *1*In_*x*_Ga_*1−x*_N/*n*GaN SLs with In content *x* = 0.25 and 0.33 in comparison with *x* = 1.

Figure 8 shows the OS ratio of SL and bulk GaN for different structures of *1*In_*x*_Ga_*1−x*_N/*n*GaN SLs with In content *x* = 0.33, *x* = 0.25 and *x* = 1. We observe that the OS increases with decreasing In content in the QW, reaching strength ratio for *1*In_*0*.*25*_Ga_*0.75*_N/*1*GaN SL equal almost 0.9. Also, the character of the oscillator strength dependence on barrier thickness changes drastically. Contrary to *1*InN/*n*GaN SLs, in the *1*In_*0*.*33*_Ga_*0.67*_N/*n*GaN SL the dependence on layer thickness is quite weak for thin barriers and does not saturate so fast. To show it more clearly we performed analogical calculations for SLs with *x* = 0.25 and we observe further tendency to weakening of the dependence on barrier thickness, in particular, the 1/1 and 1/3 SLs are characterized by the almost the same OS. It can be explained by much stronger penetration of well wave functions into the barrier region (especially for thin barriers) than could occur in the case of pure InN/GaN SLs and also by lower strength of the internal electric fields.

Concluding, based on first principles calculations we discussed band gap engineering in binary *m*InN/*n*GaN SLs and in *m*In_*x*_Ga_*1−x*_N/*n*GaN SLs taking as an example *x* = 0.33. We show that the concept of In(Ga)N/GaN superlattices enables to go far beyond the limitation of *E*_*g*_ evolution realized in ternary InGaN alloys. In particular, for a given equivalent In-content a wide range of *E*_*g*_ tunability can be achieved including even band gap closing. Whereas, in ternary alloy, In_*x*_Ga_*1−x*_N, the *E*_*g*_ does not reach the values below the band gap of InN, i.e. 0.65 eV. All the main factors influencing the band gap behavior were discussed pointing on the dominant role of internal electric field and wave functions hybridization. Their contributions to band gap values and transition matrix elements were evaluated by calculations of the internal electric fields and the oscillator strengths. We demonstrated that SL effect of wave functions hybridization is dominant for narrow wells, whereas for wider wells the effect of internal electric field is more important. It is predicted theoretically, that the PL emission intensity should drop with the increasing widths of SL layers, especially with barrier thickness, as was shown for GaN/AlN SLs^19^. The results of the band gap calculations for In_*0.33*_Ga_*0.67*_N/GaN SLs were compared with the recent experimental PL data and good agreement was obtained. Unfortunately, experimental confirmation of the performed calculations for wider range of In-content in the SL barrier is presently not possible, due to relatively poor quality of SL samples with thin layers and high indium content in QW.

## Methods

The electronic structures of the nitride SLs have been analysed by selfconsistent calculations in a supercell model. Approaches based on the Local Density Approximation (LDA) to the density functional theory, with the Perdew-Zunger parameterization^20^ of the Ceperley-Alder exchange-correlation^21^ were used. The calculations were performed in two steps, applying two different computational schemes. In the first step the atomic coordinates were determined by minimization of the Hellmann-Feynmann forces. For this task we used pseudopotentials as implemented in the Vienna *Ab-initio* Simulation Package (VASP)^16^. A cutoff energy of 600 Ry for the plane wave basis set was sufficient to obtain converged results. The calculated band gaps are very similar for cutoff energies of 400 Ry and 600 Ry.

In a second step of calculations, the band structure was obtained by including a semiempirical correction for the well-established deficiency of LDA in predicting semiconductor gaps. For this we used the Linear-Muffin-Tin-Orbital (LMTO) method^22^ in a full-potential (FP) version^23^. The semi-core cation *d* states of Ga(*3d*) and In(*4d*) were included as local orbitals^24^. Further details of the LDA-LMTO calculations are given elsewhere^24,25^.

The LDA underestimates the band gaps in semiconductors, and a correction procedure is needed which not only corrects the fundamental gap, but also the dispersion of the lowest conduction band (CB) and the values of the gaps at other points of the Brillouin zone. Therefore, a more advanced than “scissors operator” correction procedure (LDA + C) has been applied in the present work, introducing at the sites of the atoms, additional external potentials of the form^26^:

4 $$V(r)={V}_{0}(\frac{{r}_{0}}{r})\exp [-{(\frac{r}{{r}_{0}})}^{2}]$$

where *V*_0_ and *r*_0_ are adjustable parameters. The potentials are sharply peaked at the nuclear positions, and they produce “artificial Darwin shifts”, i.e. they push *s*-states, which have non-vanishing density at the nuclei (*r* = 0), upwards in energy. This method for correcting the LDA band-gap errors was developed in the context of LMTO^22^ calculations^25–30^ and applied also in a pseudopotential framework^31^.

The parameters used in the external potentials are specific for the atomic species and therefore transferable in the sense that they can be determined for the binary compounds by adjusting to experimental values of gaps and subsequently be applied to systems where the two compounds are combined, as in alloys, SLs and heterojunctions and kept unchanged while composition and volume were varied^25–29^. Our optimized values of the adjusting parameters are the following: *V*_0_(In) = *V*_0_(N) = 0, *V*_0_(Ga) = 900 Ry at the atomic sites with the range parameter set to *r*_*0*_ = 0.015 a.u. for all atoms.

To determine the internal electric fields in polar structures a semi-macroscopic model is applied. It enables to analyze the electric fields in terms of contributions from the spontaneous and piezoelectric polarizations. The electric fields in the wells (*E*_*w*_) and barriers (*E*_b_) of the SL structures can be estimated from the spontaneous polarization and piezoelectric constants of the bulk well and barrier materials. The basic relations of the model are^32^:

5 $${E}_{w}={L}_{b}({P}_{b}-{P}_{w})/({L}_{w}{\lambda }_{b}+{L}_{b}{\lambda }_{w})$$

6 $${E}_{b}=-{L}_{w}{E}_{w}/{L}_{b}$$

Here *L*_*w*_ and *L*_*b*_ denote the well and barrier widths, *λ*_*w*_ and *λ*_*b*_ the static dielectric constants of the well and barrier bulk materials, and *P*_*w*_ and *P*_*b*_ denote the polarization of the well and the barrier, respectively. The polarization may be split into its spontaneous, *P*_*sp*_, and piezoelectric part, *P*_*pz*_. *P*_*sp*_ originates from the displacements of the ions of the bulk material of the layer and for alloys is given by the linear interpolation between values given in Table 1 for binaries. *P*_*pz*_ originates from the distortions due to the in-plane lattice match to the substrate and may be expressed by piezoelectric and elastic constants^33^:

7 $${P}_{pz}={2}{e}_{{31}}{e}_{xx}+{e}_{{33}}{e}_{zz}$$

8 $${\varepsilon }_{xx}=({a}_{s}-a)/a$$

9 $${\varepsilon }_{zz}=-2{c}_{{13}}{\varepsilon }_{xx}/{c}_{{33}}$$

**Table 1:** The piezoelectric coefficients, elastic constants, spontaneous polarization and dielectric constants used in the calculations of the electric fields.

	InN	GaN
*a* (Å)	3.533	3.186
*P*_*sp*_ (C/m^2^)	−0.035^33^	−0.027^33^
*λ*	15.3^34^	10.4^34^
*e*_31_	−0.59^35^	-0.44^35^
*e*_33_	1.14^35^	0.75^35^
*c*_13_	95^36^	117^36^
*c*_33_	235^36^	400^36^

Here, *a* and *a*_*s*_ are the lattice constants of the well/barrier bulk material and the substrate, respectively. The values of parameters used in the calculations are presented in Table 1.

### Data availability

The datasets generated and analysed during the current study are available from the corresponding author on reasonable request.
